# Supplementary material for: MetaRibo-Seq measures translation in microbiomes
Source: Nat Commun. 2020 Jun 29;11:3268. doi: 10.1038/s41467-020-17081-z (PMC7324362; doi:10.1038/s41467-020-17081-z)
Supplement: Supplementary file 10 — Supplementary Data 7 [file 41467_2020_17081_MOESM10_ESM.zip › File2/Confidence_High_Taxonomy/30283_out.krona.html]

Javascript must be enabled to view this page.

members
magnitude
magnitudeUnassigned
count
unassigned
taxon
rank

30283\_out

181

181
2
superkingdom

976
phylum
181

181
200643
class

order
171549
181

179
171550
family

179
239759
genus


SRS018541\_contig\_number\_5634
1118061
species
1

178
28117

SRS011134\_contig\_number\_47709SRS011239\_contig\_number\_13700SRS011302\_contig\_number\_25632SRS012273\_contig\_number\_34456SRS012969\_contig\_number\_10339SRS013521\_contig\_number\_5014SRS013638\_contig\_number\_14609SRS013800\_contig\_number\_16435SRS014287\_contig\_number\_16163SRS014412\_contig\_number\_20856SRS014534\_contig\_number\_2324SRS014948\_contig\_number\_24278SRS015264\_contig\_number\_12651SRS015663\_contig\_number\_28804SRS015782\_contig\_number\_19538SRS016095\_contig\_number\_30440SRS016381\_contig\_number\_2304SRS016897\_contig\_number\_332SRS016954\_contig\_number\_24920SRS017103\_contig\_number\_17628SRS017191\_contig\_number\_3744SRS017307\_contig\_number\_4506SRS017433\_contig\_number\_15870SRS017622\_contig\_number\_contig-100\_421.11967SRS017701\_contig\_number\_21102SRS018623\_contig\_number\_6000SRS018836\_contig\_number\_14756SRS018888\_contig\_number\_1119SRS019267\_contig\_number\_8360SRS019286\_contig\_number\_5789SRS019381\_contig\_number\_1987SRS019445\_contig\_number\_16318SRS019910\_contig\_number\_contig-100\_3695.121030SRS020271\_contig\_number\_4347SRS020508\_contig\_number\_1044SRS020622\_contig\_number\_3288SRS021219\_contig\_number\_7044SRS022071\_contig\_number\_13171SRS022137\_contig\_number\_14914SRS022524\_contig\_number\_9415SRS022609\_contig\_number\_44865SRS023526\_contig\_number\_16106SRS023971\_contig\_number\_4271SRS024331\_contig\_number\_32053SRS024435\_contig\_number\_26249SRS024625\_contig\_number\_18116SRS043701\_contig\_number\_14262SRS043768\_contig\_number\_14790SRS045004\_contig\_number\_6263SRS045195\_contig\_number\_7498SRS045244\_contig\_number\_8896SRS045645\_contig\_number\_25441SRS045713\_contig\_number\_16252SRS045826\_contig\_number\_12345SRS047044\_contig\_number\_14812SRS048060\_contig\_number\_2851SRS048164\_contig\_number\_24379SRS049712\_contig\_number\_15371SRS049773\_contig\_number\_12713SRS049896\_contig\_number\_26059SRS049995\_contig\_number\_2670SRS050026\_contig\_number\_2157SRS050299\_contig\_number\_10565SRS050422\_contig\_number\_contig-100\_3165.149362SRS050520\_contig\_number\_882SRS050941\_contig\_number\_21530SRS050998\_contig\_number\_7717SRS051031\_contig\_number\_31453SRS052576\_contig\_number\_48SRS053398\_contig\_number\_11177SRS053649\_contig\_number\_16619SRS054059\_contig\_number\_contig-100\_78.48781SRS055982\_contig\_number\_10667SRS057480\_contig\_number\_9244SRS058145\_contig\_number\_contig-100\_462.35065SRS058770\_contig\_number\_16258SRS062701\_contig\_number\_5930SRS063040\_contig\_number\_35361SRS063489\_contig\_number\_5720SRS063518\_contig\_number\_38004SRS065176\_contig\_number\_9820SRS074670\_contig\_number\_8788SRS075773\_contig\_number\_13592SRS075821\_contig\_number\_20093SRS075878\_contig\_number\_14770SRS075963\_contig\_number\_199SRS075984\_contig\_number\_17301SRS076976\_contig\_number\_13491SRS077024\_contig\_number\_10702SRS077086\_contig\_number\_778SRS077392\_contig\_number\_17183SRS077641\_contig\_number\_2536SRS077849\_contig\_number\_contig-100\_1253.167252SRS078176\_contig\_number\_12876SRS078242\_contig\_number\_22611SRS078665\_contig\_number\_8038SRS097920\_contig\_number\_21906SRS097958\_contig\_number\_17694SRS098061\_contig\_number\_2173SRS098571\_contig\_number\_57298SRS098620\_contig\_number\_contig-100\_17083.17083SRS098655\_contig\_number\_9010SRS100907\_contig\_number\_8670SRS101376\_contig\_number\_1774SRS101433\_contig\_number\_8565SRS103987\_contig\_number\_6339SRS104036\_contig\_number\_4017SRS1041033\_contig\_number\_20265SRS1041037\_contig\_number\_15829SRS1041092\_contig\_number\_406SRS1041130\_contig\_number\_5119SRS1041133\_contig\_number\_19892SRS1041134\_contig\_number\_8951SRS1041141\_contig\_number\_14361SRS1041142\_contig\_number\_6670SRS1041143\_contig\_number\_3337SRS1041145\_contig\_number\_25429SRS1041147\_contig\_number\_2453SRS1041157\_contig\_number\_6664SRS1041164\_contig\_number\_contig-100\_1646.28455SRS104327\_contig\_number\_1721SRS104636\_contig\_number\_22096SRS104912\_contig\_number\_7539SRS104975\_contig\_number\_3756SRS105082\_contig\_number\_15754SRS105153\_contig\_number\_800SRS1054691\_contig\_number\_11601SRS1054716\_contig\_number\_15580SRS1055022\_contig\_number\_24781SRS1055034\_contig\_number\_9773SRS1055038\_contig\_number\_5438SRS1055069\_contig\_number\_3314SRS1055076\_contig\_number\_10638SRS1055083\_contig\_number\_1989SRS1055095\_contig\_number\_498SRS140513\_contig\_number\_1336SRS142542\_contig\_number\_4783SRS142599\_contig\_number\_25990SRS143148\_contig\_number\_23041SRS143342\_contig\_number\_12107SRS143372\_contig\_number\_7233SRS143523\_contig\_number\_1330SRS143876\_contig\_number\_37294SRS143895\_contig\_number\_29007SRS144135\_contig\_number\_22416SRS144297\_contig\_number\_13197SRS144362\_contig\_number\_13391SRS144506\_contig\_number\_42143SRS144603\_contig\_number\_4971SRS144714\_contig\_number\_25433SRS145308\_contig\_number\_contig-100\_328.37702SRS145497\_contig\_number\_3310SRS146812\_contig\_number\_48747SRS147139\_contig\_number\_12110SRS147346\_contig\_number\_17916SRS147425\_contig\_number\_11140SRS148196\_contig\_number\_17853SRS148253\_contig\_number\_3545SRS148784\_contig\_number\_12921SRS148817\_contig\_number\_565SRS148874\_contig\_number\_740SRS149075\_contig\_number\_16368SRS149181\_contig\_number\_1970SRS149784\_contig\_number\_25746SRS149879\_contig\_number\_contig-100\_15270.65185SRS892882\_contig\_number\_10779SRS893170\_contig\_number\_10793SRS893172\_contig\_number\_9680SRS893187\_contig\_number\_17547SRS893230\_contig\_number\_contig-100\_86.80889SRS893252\_contig\_number\_19325SRS893279\_contig\_number\_1458SRS893358\_contig\_number\_6838SRS893369\_contig\_number\_9860SRS893378\_contig\_number\_15501SRS893380\_contig\_number\_1272SRS893382\_contig\_number\_2704SRS971275\_contig\_number\_41472
species

815
family
2

816
genus
2

1
821

SRS019808\_contig\_number\_2093
species


SRS018133\_contig\_number\_4524
46506
species
1
